# Aging in nucleus accumbens and its impact on alcohol use disorders

**DOI:** 10.1016/j.alcohol.2022.08.004

**Published:** 2022-09-07

**Authors:** Macarena Konar-Nié, Alejandra Guzman-Castillo, Lorena Armijo-Weingart, Luis Gerardo Aguayo

**Affiliations:** a Laboratory of Neurophysiology, Department of Physiology, Universidad de Concepcion, Concepcion, Chile; b Programa en Neurociencia, Psiquiatría y Salud Mental, Universidad de Concepción, Concepcion, Chile

**Keywords:** Aging, Alcohol abuse disorders, Limbic system, Neurotransmitters, Nucleus accumbens, Receptors

## Abstract

Ethanol is one of the most widely consumed drugs in the world and prolonged excessive ethanol intake might lead to alcohol use disorders (AUDs), which are characterized by neuroadaptations in different brain regions, such as in the reward circuitry. In addition, the global population is aging, and it appears that they are increasing their ethanol consumption. Although research involving the effects of alcohol in aging subjects is limited, differential effects have been described. For example, studies in human subjects show that older adults perform worse in tests assessing working memory, attention, and cognition as compared to younger adults. Interestingly, in the field of the neurobiological basis of ethanol actions, there is a significant dichotomy between what we know about the effects of ethanol on neurochemical targets in young animals and how it might affect them in the aging brain. To be able to understand the distinct effects of ethanol in the aging brain, the following questions need to be answered: (1) How does physiological aging impact the function of an ethanol-relevant region (e.g., the nucleus accumbens)? and (2) How does ethanol affect these neurobiological systems in the aged brain? This review discusses the available data to try to understand how aging affects the nucleus accumbens (nAc) and its neurochemical response to alcohol. The data show that there is little information on the effects of ethanol in aged mice and rats, and that many studies had considered 2–3-month-old mice as adults, which needs to be reconsidered since more recent literature defines 6 months as young adults and >18 months as an older mouse. Considering the actual relevance of an aged worldwide population and that this segment is drinking more frequently, it appears at least reasonable to explore how ethanol affects the brain in adult and aged models.

## Introduction

Ethanol is one of the most widely consumed drugs in the world, and prolonged excessive ethanol intake might lead to alcohol use disorders (AUDs) which are characterized by neuroadaptations in different brain regions, such as in the reward circuitry (Bahi, 2020; Bahi & Dreyer, 2019; Griffin, Haun, Hazelbaker, Ramachandra, & Becker, 2014; Jonsson, Ericson, & Söderpalm, 2014). Nevertheless, the neurobiological mechanisms involved in AUDs are still not well understood (Jeanes, Buske, & Morrisett, 2011, 2014; Abrahao et al., 2013; Bahi & Dreyer, 2012).

There is a significant dichotomy between what we know about the effects of ethanol on neurochemical targets in young animals and how it might affect them in the aging brain. In addition, the global population is aging, and it appears that they are increasing their ethanol consumption. To be able to understand the distinct effects of ethanol in the aging brain, we need to answer the following questions: (1) How does physiological aging impact the function of an ethanol-relevant region (e.g., the nucleus accumbens)? (2) What are the effects of ethanol on defined neurobiological endpoints (e.g., glutamatergic neurotransmission) at an early stage? and (3) How does ethanol affect these neurobiological systems in the aged brain? This review will discuss the available data to try to understand how aging affects the nucleus accumbens (nAc) and its neurochemical response to alcohol.

At the onset of this review, we should define the different stages of aging in rodents, primarily C57BL/6J mice, using a proposed guideline for chronological age (months) (Flurkey, Currer, & Harrison, 2007). An adolescent animal is <3 months of age, a young adult corresponds to 3–6 months, a 12-month-old relates to a middle-aged adult, and an aged (old) mouse is 22–28 months. More importantly, as a general guideline to humans, these ages were compared to mature (20–30 years), middle age (38–47 years), and old (>56 years). Interestingly, most studies with ethanol have used 2–3-month-old mice, corresponding to late adolescent–young adults, when the brain is still undergoing growth and development (Bandeira, Lent, & Herculano-Houzel, 2009; Fu, Rusznak, Herculano-Houzel, Watson, & Paxinos, 2013). In mice and rats that are commonly used in biomedical research, it is not straightforward to place the boundary between adolescence from adulthood, but it is in the neighborhood of 2.5 months (Spear, 2016). Therefore, as recently recommended by a large survey of mice ages used in biomedical research and the fact that some features of brain development are still in progress, 12–16-week-old mice should be the minimum age for an adult model (Jackson et al., 2017). This age appears to also define the plateau of development (late adolescence) and the onset of adulthood or brain maturity based on structure and functional connectivity (Egimendia et al., 2019; Kerkenberg et al., 2021), behavior, synaptic structure and plasticity, and calcium homeostasis (Radulescu, Cerar, Haslehurst, Kopanitsa, & Barnes, 2021). In addition, another recent study showed ongoing neurogenesis and increasing myelination in mice up to 6 months of age (Hammelrath et al., 2016).

## Alcohol abuse disorders and aging

Alcohol consumption is a common practice in older adults with a prevalence exceeding 50% (Blazer & Wu, 2009; Breslow, Castle, Chen, & Graubard, 2017; Laberge, Bigelow, Lagarde, & Crizzle, 2020). Although average alcohol consumption declines with age (R. Molander, Yonker, & Krahn, 2010), recent studies have reported that older adults exhibit a greater increase in consumption in later years (Breslow et al., 2017; Keyes et al., 2019), including heavy drinkers (B. H. Han, Moore, Sherman, Keyes, & Palamar, 2017; Immonen, Valvanne, & Pitkala, 2011). In addition to average consumption measurements, other aspects of consumption such as frequency and amount consumed per drinking event, have also shown significant differences with age. For example, age-dependent increases in drinking frequency and decreases in the amount of alcohol consumed per event have been observed (Lewis, Garcia, & Nixon, 2018; Molander et al., 2010).

Given the high prevalence of alcohol consumption and the changes in drinking patterns described in older adults, it is critical to understand how aging influences the effects of alcohol. In order to distinguish how the age-related effects of alcohol occur, it is important to remember that the nervous system undergoes neuroplastic structural and functional changes during aging. Main findings highlight a loss of volume and an increase in ventricular cavities; however, the pattern of change is not homogeneous (Fjell & Walhovd, 2010; Sele, Liem, Mérillat, & Jäncke, 2020; Tamnes et al., 2013).

The effects of alcohol on the nervous system have been well-described in adult humans and rodents. Alcohol acts as a central nervous system depressant having anxiolytic, sedative, and memory-disrupting properties (Eckardt et al., 1998). Additionally, it is known that alcohol produces marked alterations in learning and memory processes (White, 2003). Although research involving the effects of alcohol in aging subjects is limited, differential neurobiological effects have been described, even at low doses of alcohol. For example, studies in humans show that older adults perform worse in tests assessing working memory, attention, and cognition as compared to younger adults (Garcia, Lewis, Boissoneault, & Nixon, 2020; L. Han & Jia, 2021; Lewis, Boissoneault, Frazier, & Nixon, 2016; Price, Lewis, Boissoneault, Frazier, & Nixon, 2018). Regarding studies in animal models, the main effects of alcohol that have been evaluated are cognition, ataxia, hypothermia, and sedation. In the latter three, an age-dependent increase was observed when comparing adolescent, adult, and aged animals (Matthews et al., 2019; Ornelas, Novier, Van Skike, Diaz-Granados, & Matthews, 2015; Perkins, Vore, Lovelock, Varlinskaya, & Deak, 2018; Silveri & Spear, 2000; Van Skike et al., 2010; Watson, James, Mittleman, & Matthews, 2020). In studies where the effects on cognition were tested, specifically spatial memory tests, no differences were observed between adolescent and adult animals following an acute dose of alcohol (Chin et al., 2011; Matthews, Scaletty, Schreiber, & Trapp, 2020); however, aged animals did show impairment on memory tasks in general, both those assessing spatial and non-spatial memory (Matthews et al., 2020).

Despite the above, it is likely that aging is associated with psychosocial changes that could influence drinking characteristics (Han, Moore, Ferris, & Palamar, 2019; Han et al., 2017; Wang, Zhang, & Wu, 2022). However, given the changes occurring in the aging brain and the differential effects of alcohol in both human and animal models, it is likely that some of the underlying neurobiological bases are in changes in the reward system, i.e., a decrease in dopamine signaling, synaptic plasticity, and excitability.

## Overall brain aging

Aging is a physiological process that involves a series of structural, neurochemical, and functional changes that might cause progressive alterations in cellular and circuit control and integration (Burke & Barnes, 2006; Ouda, Profant, & Syka, 2015). Age-related changes might result in loss of memory, sensory perception, motor coordination, cognition, working memory, and executive functions (Alexander, Mushtaq, Osmond, & Amoaku, 2012; Dykiert, Der, Starr, & Deary, 2012; Grau-Perales, Gomez-Chacon, & Gallo, 2019; Hahr, 2019; Noda, Sato, Fukuda, Tada, & Hattori, 2020; Ouda et al., 2015). These losses might depend on factors such as lifestyle, diet, and social behaviors (Samarakoon, Chandola, & Ravishankar, 2011). For example, it is known that aging affects the normal function of various brain regions such as the hippocampus (HC) and the prefrontal cortex (PFC), and these have been extensively studied (Chang, Rosene, Killiany, Mangiamele, & Luebke, 2005; Kelly et al., 2006; Morrison & Baxter, 2012; Rosenzweig, Redish, McNaughton, & Barnes, 2003). A less studied region in the context of aging is the nucleus accumbens, a critical region of the reward system that also participates in motivation (Collins, Aitken, Greenfield, Ostlund, & Wassum, 2016; Schall, Wright, & Dong, 2020; Soares-Cunha et al., 2016).

## Age-related changes in the nucleus accumbens

The nucleus accumbens (nAc) is a major component of the ventral striatum and a critical region of the reward system involved in cognition, emotion, and addiction-related behaviors (Floresco, 2015; Radke et al., 2019; Russo & Nestler, 2013). The nAc is composed mainly of GABAergic medium spiny neurons (MSNs) and receives an important dopaminergic connection from the VTA, cholinergic projections from the lateral septum, and glutamatergic input from the amygdala, hippocampus, paraventricular nucleus, and insular cortex (Gehrlach et al., 2020; Humphries & Prescott, 2010; Jaramillo, Van Voorhies, Randall, & Besheer, 2018; Sesack & Grace, 2010). Several studies have suggested that the nAc plays a crucial role in action goal-seeking behavior (Floresco, 2015; Radke et al., 2019; Russo & Nestler, 2013). It also integrates cognitive and affective information from different brain regions to increase the efficacy and strength of appetitive or aversive motivated behaviors (Hikida, Kimura, Wada, Funabiki, & Nakanishi, 2010; Klawonn & Malenka, 2018; Nakanishi, Hikida, & Yawata, 2014; Yang et al., 2018). Additionally, dopaminergic inputs originating from the VTA are a key component in the modulation of nAc activity (Nakanishi et al., 2014). Thus, dopamine (DA) is recognized as a key neurotransmitter involved in the reinforcing properties of ethanol (Bahi & Dreyer, 2012; Dahchour & Ward, 2022; Liu, Liu, et al., 2020).

In this review, we will discuss the available evidence pointing to structural and functional changes in the nAc during aging. Importantly, since the average life expectancy is increasing, these changes may help to understand neurobiological underpinnings for age-related behavioral alterations, such as cognitive decline, disturbed emotional processing, difficulty with social interaction, and higher prevalence in AUDs during aging (Alejandro Borja, Alejandro Navarro, Beatriz, Ignacio, & Milagros, 2020; Buck, Torregrossa, Logan, & Freyberg, 2021; Crimmins, 2015; Kogias et al., 2020; Morrison, McGinty, du Hoffmann, & Nicola, 2017; Oeppen & Vaupel, 2002; Richter, Reinhard, Kraemer, & Gruber, 2020; Zhang et al., 2020; Zhu, Ottenheimer, & DiLeone, 2016). Specifically, the nAc plays a critical role in reward, locomotion, memory, motivation, aversion, social interactions, and addictive behaviors (i.e., ethanol) (Alejandro Borja et al., 2020; Bond et al., 2020; Borrego et al., 2022; Fox, Figueiredo, Menken, & Lobo, 2020; Freels, Gabriel, Lester, & Simon, 2020; Guadarrama-Bazante & Rodriguez-Manzo, 2019; Hurley & Carelli, 2020; Ji et al., 2017; Lee et al., 2020; Liu, Montgomer et al., 2020; Purohit et al., 2018; Richter et al., 2020; Wang et al., 2019; Zhang et al., 2020; Zhu et al., 2016), and these are altered during aging (Bordner et al., 2011; Churchwell & Yurgelun-Todd, 2013; Dowling, Weiss, & Condon, 2008; Harb, Sousa, Zihl, & Almeida, 2014; Moron & Gallo, 2007; Samanez-Larkin, Kuhnen, Yoo, & Knutson, 2010; Schott et al., 2007; Stulhofer, Hinchliff, Jurin, Carvalheira, & Traeen, 2019; Uryash, Flores, Adams, Allen, & Lopez, 2020; Wu, Schimmele, & Chappell, 2012; Zhaoyang, Sliwinski, Martire, & Smyth, 2018). The shell and the core of the nAc are important in reward and ethanol addictive behavior, respectively (Al-Muhtasib, Forcelli, & Vicini, 2018; Meredith, Agolia, Arts, Groenewegen, & Zahm, 1992; Purohit et al., 2018; Renteria, Buske, & Morrisett, 2018; Wright, Beijer, & Groenewegen, 1996; Zaborszky et al., 1985), and this will be discussed in terms of brain aging.

### Volume loss

A previous finding showed a significant reduction in human brain weight during aging (Svennerholm, Bostrom, & Jungbjer, 1997). Interestingly, cross sectional and longitudinal analysis suggests an age-associated decrease in global and regional brain volume (Scahill et al., 2003; Trollor & Valenzuela, 2001; Yoo, De Ridder, & Vanneste, 2016). Particularly, the nAc appears to be significantly affected in human aging (Jernigan et al., 2001; Liu, Liu, et al., 2020; Pressman et al., 2016; Walhovd et al., 2005, 2011; Wan et al., 2020; Yoo et al., 2016). For instance, functional imaging studies showed a significant reduction in the nAc volume (about 40%) at a 0.4% rate per year (Fjell & Walhovd, 2010). Moreover, a more recent advanced brain volumetric analysis, combining magnetic resonance imaging (MRI) with fractal analysis, supports a significant reduction of nAc volume with age (Lahmiri, 2016; Liu, Liu, et al., 2020).

### Neuronal loss

Previous work suggested that normal aging was associated with a massive and global neuronal loss throughout the brain (Alvarez et al., 1998; Ball, 1977; Coleman, Flood, & West, 1987; Cruz-Sanchez, Moral, Tolosa, de Belleroche, & Rossi, 1998; Mani, Lohr, & Jeste, 1986; Mortera & Herculano-Houzel, 2012). However, recent studies applying new stereological principles for cell counting have challenged this idea (Burke & Barnes, 2006). For example, analysis and quantification of nAc neurons from coronal brain sections obtained from post mortem healthy humans found no significant difference between 35- and 65-year-old individuals (Huang & Zhao, 1995). Therefore, the available evidence seems to indicate that healthy nAc aging is not associated with major neuronal loss. However, in the absence of a large neuronal loss, it is possible that microdomain alterations in excitability and neurotransmission cause alterations in accumbal functions (Cepeda, Lee, Buchwald, Radisavljevic, & Levine, 1992; Walsh & Akopian, 2019). Thus, the functional disruption of these neural networks involved in motivation, cognition, or motor alterations could partly explain the behavioral changes observed in aging.

### Neuronal morphology changes with age

Since neuronal loss in the nAc during normal aging is not marked (K. W. Huang & Zhao, 1995), changes in microstructures, such as branching and synaptic transmission, could provide an explanation for the volume reduction reported in this limbic structure (Jernigan et al., 2001; Liu, Liu, et al., 2020; Narvacan, Treit, Camicioli, Martin, & Beaulieu, 2017; Pressman et al., 2016; Walhovd et al., 2005, 2011; Wan et al., 2020). During aging, neurons undergo morphological alterations such as dendritic tree modifications that could affect neurotransmission (Hahr, 2019). For example, studies carried out on striatal neurons using a Golgi analysis method that allows evaluating geometric characteristics of the dendritic spines of neurons demonstrated dendritic atrophy and loss of dendritic spines in MSNs of aged animals (Levine et al., 1986; Walsh & Akopian, 2019). On the other hand, it remains to be determined whether a reduction in neurotransmission during aging could modify the morphology of nAc neurons. For instance, it has been shown that DA depletion in young rats alters the morphology of MSNs from the dorsal striatum. Thus, rats with reduced dopamine levels showed significantly shorter dendrites and fewer spines compared to controls, suggesting that DA levels might be an important regulator of neuronal morphology (Azdad et al., 2009). Importantly, several studies reported that DA concentrations are significantly decreased in aged rodents (Huang, Wang, Tai, Tsai, & Peng, 1995; Segovia, Del Arco, & Mora, 1999; Winner et al., 2017). Hence, reduction in the levels of this neurotransmitter could mediate changes in accumbal MSNs morphology. For example, it has been reported that in Alzheimer’s disease the loss of dendritic spines caused by the β-amyloid peptide (Aβ) results in the fragmentation of the cortical network (Kashyap et al., 2019). Could this effect be somewhat similar to healthy aging? Additional studies are necessary to examine whether age-related neurotransmitter depletion alters the morphology and functionality of accumbal MSNs.

## Main neurotransmitters affected by aging

**Fig. 1.**
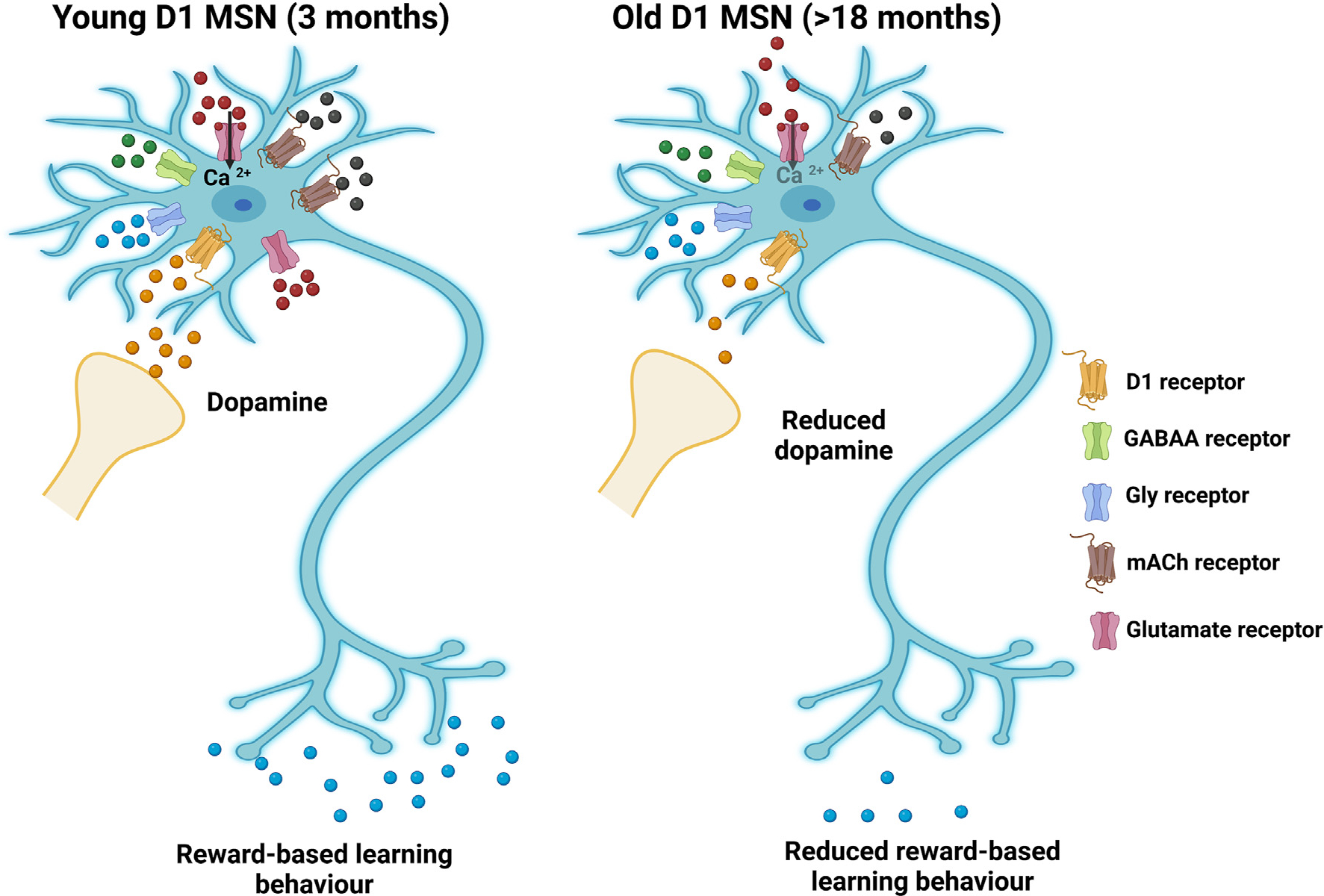
Changes in diverse neurotransmissions in nAc neurons during aging. GABAergic medium spiny neurons (MSNs) represent more than 90% of neurons in the nAc. Young MSNs receive a variety of inputs from diverse regions involved in specific patterns of behaviors. Neurotransmitter functions and dopamine levels in older nAc are altered leading to reduced excitability and altered reward learning behaviors. Created with BioRender.com.

The proper functioning of neural networks, as well as the interactions through chemical synapses, is mainly regulated by neurotransmitters (NTs) (Hampel & Lau, 2020). The level and functions of NTs in the nAc have been evaluated with different techniques (Huang & Zhao, 1995; Segovia & Mora, 2005; Winner et al., 2017). Although there are limited data on NTs alterations in the nAc during aging, we will discuss some studies supporting the idea that the neurochemical environment in the nAc changes during aging. For instance, no significant differences have been found between young and older animals regarding their GABA or glutamate levels in the nAc (see Table 1) (Segovia et al., 1999; Segovia & Mora, 2005). Although glycine and acetylcholine have been reported to be present in the accumbens, their levels have not been evaluated in the aged brain (Collins et al., 2016; Muñoz, Yevenes, Forstera, Lovinger, & Aguayo, 2018). On the other hand, several lines of evidence support a significant reduction of DA levels in the nAc from the aged brain (see Fig. 1) (Huang & Zhao, 1995; Winner et al., 2017). For instance, a recent study using high pressure liquid chromatography coupled with electrochemical detection (HPLC-ED) measured the basal level of DA in the nAc of young (3–8 months) and aged mice (22–28 months) and found that accumbal DA levels decreased in the aged mice (Winner et al., 2017). These results are in agreement with a previous study by Huang et al. (1995) (see Table 1). Nevertheless, no significant changes have been observed in the level of the DA transporter (DAT) and vesicular monoamine transporter 2 (VMAT2) between young and old mice (Karrer, Josef, Mata, Morris, & Samanez-Larkin, 2017; Winner et al., 2017).

Interestingly, Winner et al. (2017) also showed that old mice have lower concentrations of DOPAC in the nAc than young mice. DOPAC arises from the metabolism of recaptured DA in presynaptic terminals (Roth, 1976). In this way, DOPAC represents an indirect index of DA that has been released and recaptured by the presynaptic neuron. The reduced DOPAC levels in the aged nAc could suggest a decrease in dopaminergic neuronal firing activity that translated into less DA release and therefore less DOPAC metabolism (Winner et al., 2017). Nevertheless, these results suggest that the reduction in dopamine levels in the nAc might contribute to reduced motivation, reward, and spontaneous locomotor activity found in older individuals (Crawford & Levine, 1997; Hebert & Gerhardt, 1998; Huang et al., 1995).

On the other hand, the functional interaction between different NTs and DA is well recognized (Imperato, Honoré, & Jensen, 1990; Nisell, Nomikos, & Svensson, 1994; Pitman, Puil, & Borgland, 2014; Saigusa et al., 2012; Saul’skaya, Mikhailova, & Gorbachevskaya, 2001; Sesack & Pickel, 1990; Taepavarapruk, Floresco, & Phillips, 2000), and aging alters the strength of these functional interactions (Umegaki, Roth, & Ingram, 2008). For example, glutamate regulates extracellular levels of dopamine and GABA in the nAc (Segovia & Mora, 2005). Consequently, local perfusion of ionotropic glutamate receptor agonists into the nAc of young rats increases extracellular DA concentrations, whereas this effect is significantly attenuated in middle-aged and elderly rats (Segovia & Mora, 2005). In contrast, the glutamate-induced increase in GABA release is greater in the nAc from aged rats as compared with young animals, suggesting that a reduced dopamine release, together with an increment in GABA in response to glutamatergic inputs, converge to a change in the excitation-inhibition ratio in the nAc of aged animals. These changes might explain the reduction in ventral striatum activation in older subjects in response to a previous reward signal and also a decreased spontaneous locomotor activity in aged rats (Dreher, Meyer-Lindenberg, Kohn, & Berman, 2008; Hebert & Gerhardt, 1998; Huang et al., 1995).

Interestingly, it was reported that application of a dopamine D2 receptor (D2R) agonist into the striatum decreased acetylcholine levels in young (6 months) and old rats (24 months), with a greater effect in older rats (Umegaki et al., 2008). This age-related decline could lead to impairments in motor performance common in aging (Frolov et al., 2020; Umegaki et al., 2008). However, alterations in DA modulation of acetylcholine levels during aging in the ventral striatum have not been addressed. On the other hand, studies demonstrated a functional interaction between dopamine and glycine. For instance, dopamine decreases glycine levels in the nAc during food consumption (Saul’skaya et al., 2001), and glycine regulates dopamine release in the same regions after ethanol administration in rats (Molander & Söderpalm, 2005a; 2005b). However, the effect of aging on this cross modulation has not been examined yet.

### Age-related changes in neurotransmitter receptor expression

Brain aging and neurotransmission alterations are widely linked to changes in the expression and function of key neurotransmitter receptors (de Oliveira, Ramos, Amaro, Dias, & Vieira, 2019; Utkin, 2019). These complexes regulate neuronal activity either through the opening of ion channels (ionotropic receptors) or the generation of second messengers (metabotropic receptors), thus playing a key role in intercellular connectivity of the nervous system (Hampel & Lau, 2020). Therefore, in this section we will review age-related changes in neurotransmitter receptor expression (see Table 2, Fig. 1).

### Dopamine receptors

The MSNs in the nAc are subdivided into two different types: those that preferentially express dopamine type D1 receptors (D1R; Gs coupled) and are part of the direct pathway, and those that express dopamine D2 type receptors and are part of the indirect pathway (D2R; Gi coupled) (Klawonn & Malenka, 2018; Russo & Nestler, 2013). Studies suggest that each of these pathways have different roles. For instance, the direct pathway is mainly associated with reward, whereas the indirect pathway is likely related to aversive behavior (Gallegos, Muñoz, Araya, & Aguayo, 2019; Hikida et al., 2010, 2013; Nakanishi et al., 2014). Importantly, alterations in the dopaminergic system during physiological aging are widely recognized (Backman, Lindenberger, Li, & Nyberg, 2010; Kaasinen & Rinne, 2002). Studies showed a decrease in the density of D1R, as well as a reduction in receptor binding by its agonist in the striatum of elderly individuals (Rieckmann et al., 2011; Suhara et al., 1991; Y. Wang et al., 1998; L. Zhang & Roth, 1997). However, the effects of aging on D1R have been poorly studied in the nAc, despite the wide expression of these receptors in accumbal MSNs (Soares-Cunha et al., 2018, 2020). Positron emission tomography (PET) measurements using a D1R radioligand [^11^C] SCH23390 in the nAc reported a 17% decrease in D1R binding in elderly individuals (70 years old) compared to young individuals (25 years old) (Rieckmann et al., 2011). In contrast, more recent PET measurements, using the same radioligand in mice, indicated that D1R binding did not exhibit significant changes in 24-month-old mice compared to young animals (Giacobbo et al., 2022). Another radioligand study in aged rats showed that the density of D2R in the accumbens was not altered with aging (Araki, Kato, Shuto, Fujiwara, & Itoyama, 1997). However, analysis with [^18^F] fallypride-PET showed that this receptor exhibits a decrease in the binding to its ligand in the nAc of older individuals (Dang et al., 2017). Consistently, a recent study using PET showed a significant reduction in D2R binding in the nAc of aged C57BL/6J mice (Giacobbo et al., 2022). Finally, the expression of the dopamine 3 receptor, D3R, is susceptible to changes during aging. Autoradiography studies showed that D3R density increased in aged Fischer 344 × Brown-Norway rats (Wallace & Booze, 1996). On the contrary, human studies showed that D3R was not modified during aging (Matuskey et al., 2016; Nakajima et al., 2015). Thus, it is possible that the increase in D3R expression could reflect a compensatory reaction in response to loss of accumbal D2R. These alterations in the dopaminergic pathway could explain the alterations in learning and motor activity during aging (Backman et al., 2010). These differences highlight the difficulties of comparing animal models with human data and the presence of basal differences.

### Glutamate receptors

Ionotropic glutamate receptors (iGluRs) are tetrameric nonselective cation channels that mediate excitatory neurotransmission in the central nervous system (Sobolevsky, 2015; Twomey & Sobolevsky, 2018). Glutamate is the primary neurotransmitter that binds to postsynaptic iGluRs, thus inducing depolarization of the membrane, and initiating signal transduction in the postsynaptic neuron (Sobolevsky, 2015; Twomey & Sobolevsky, 2018). There are three major classes of iGluRs: N-methyl-d-aspartate (NMDA), α-amino-3-hydroxy-5-methyl1–4-isoxazolepropionoc acid (AMPA), and Kainate receptors. Among iGluRs, NMDA and AMPA receptors have been the most extensively studied, and they are affected by ethanol (Abrahao et al., 2013; Guidi, Kumar, & Foster, 2015; Henley & Wilkinson, 2013; Jurado, 2017; Kumar, 2015; Kumar & Foster, 2019; Magnusson, 2012; Newcomer, Farber, & Olney, 2000; Pandey, Rai, Gaur, & Prasad, 2015; Segovia, Porras, Del Arco, & Mora, 2001). NMDA receptors are both voltage- and ligand-dependent channels that form hetero-tetrameric complexes composed of different subunits (GLUN1, GLUN2A-D, and GLUN3A-B). Calcium entry to the postsynaptic neurons through NMDARs channels induces AMPARs mobilization and synaptic remodeling, thus strengthening or weakening the synaptic events (Kumar & Foster, 2019; Turner, Kashima, Manz, Grueter, & Grueter, 2018).

Several studies in the hippocampus and cortex showed that NMDARs and AMPARs become significantly hypofunctional with aging (from 18-month-old mice and 14-month-old rats) (Guidi et al., 2015; Henley & Wilkinson, 2013; Jurado, 2017; Kumar & Foster, 2013, 2019; Mora, Segovia, & Del Arco, 2008; Newcomer et al., 2000; Pandey et al., 2015; Segovia & Mora, 2005; Segovia et al., 2001; Wenk & Barnes, 2000; Zhao et al., 2009) (see Fig. 1). Importantly, synaptic plasticity is a key component for learning and memory; thus, reduced function of these receptors likely accounts for age-related cognitive decline (Guidi et al., 2015; Kumar, 2015; Kumar & Foster, 2013; Newcomer et al., 2000; Zhao et al., 2009). For instance, impaired memory and learning in 26-month-old mice and 14–18-month-old rats are associated with reduced NMDARs function and expression in the hippocampus and cortex (Guidi et al., 2015; Kumar, 2015; Kumar & Foster, 2013, 2019; Magnusson, 2012; Wenk & Barnes, 2000; Zhao et al., 2009). Furthermore, a study done in 25–29-month-old rats suggested that impaired synaptic plasticity in the hippocampus during normal aging might also be attributed to defects in the trafficking and function of AMPARs (Jurado, 2017). In addition, GluR2 subunit-containing AMPARs play an important role in synaptic plasticity, and their expression is significantly reduced in 18-month-old mice (Pandey et al., 2015). Studies in the nAc showed a diminished binding of [3H]MK-801 (an NMDAR open channel blocker) and [3H]-AMPA in 18-month-old rats, suggesting reduced NMDARs and AMPARs density with age (Ossowska et al., 2001). Consistently, the increase in extracellular dopamine in the nAc induced by activation of these receptors was significantly reduced in aged rats (Mora, Segovia, & Del Arco, 2008; Segovia & Mora, 2005). Regarding metabotropic glutamate receptors, it was found that the mRNA expression of the mGluR3 receptor increased in the accumbal core in 25-month-old Fisher rats (Simonyi et al., 2005). Because glutamatergic inputs to the nAc modulate motor activity, alterations in accumbal glutamate receptors could contribute to deficits characteristic of aging (Ossowska et al., 2001; Svensson, Zhang, Johannessen, & Engel, 1994; Taepavarapruk et al., 2000). Overall, the data show that global striatum is affected by aging, which would explain motor deficits. However, the mechanisms by which alterations in the nAc might affect motivation, mood, and reward are largely unknown.

### Acetylcholine receptors

Acetylcholine receptors (AChRs) in the nAc participate in the modulation of dopamine release, and therefore, in motivation and acquisition of drug reinforcement (Collins et al., 2016; Crespo, Sturm, Saria, & Zernig, 2006). Importantly, acetylcholine muscarinic (mAChR) and nicotinic (nAChR) receptors are broadly distributed in the nAc (Cachope et al., 2012; Threlfell et al., 2010), and medium spiny neurons express M1 and M4 muscarinic receptors (Yan, Flores-Hernandez, & Surmeier, 2001). Age-associated changes in muscarinic receptors in the nAc have been reported. For example, *in vitro* autoradiography experiments showed a significant decline in [3H]-quinuclidinyl benzilate, mAChR ligand binding in the nAc of old rats (24–25 months). No evidence of mAChR mRNA alterations was observed in aged rodents, thus suggesting that reduced mAChR density was not related to a decline in receptor gene expression (Blake et al., 1991). Age-related alterations of nicotinic acetylcholine receptors in the brain are widely recognized (Utkin, 2019). However, these studies have been carried out in cortical regions and focus on pathological aging (Gahring, Persiyanov, Days, & Rogers, 2005; Ghimire, Cai, Ling, Hackett, & Caspary, 2020; Picciotto & Zoli, 2002), and the effect of aging on accumbal nicotinic receptors is still unknown. AChRs in the nAc play an essential role in supporting the excitability and neurotransmission of this network; therefore, a comprehensive study on their potential modification with age is needed.

### Inhibitory receptors

γ-Aminobutyric acid (GABA) type A receptors (GABA_A_Rs) and glycine receptors (GlyRs) are chloride-permeable pentameric LGICs (ligand-gated ion channels) that mediate fast inhibitory synaptic neurotransmission in the CNS (Gielen & Corringer, 2018). Impaired inhibitory signaling within cortical circuits is an important feature in different psychiatric and neurodegenerative diseases, highlighting a key role of these receptors in the maintenance of the nervous system network (Chang & Martin, 2011; Comhair et al., 2018; Eichler et al., 2008; Gielen & Corringer, 2018; Lemoine et al., 2012).

### Glycine receptors

GlyRs are critical in respiratory and motor control, and pain perception, among other biological functions (Legendre, 2001; Lynch, Zhang, Talwar, & Estrada-Mondragon, 2017). Diversity of GlyRs results from differential expression of four α subunits (α1–4) and one β subunit. GlyRs can form homo- (5α) or hetero-pentameric receptors (combination of α and β subunits). The α subunits contain the binding site for agonists (i.e., glycine) and antagonists (i.e., strychnine) and are also responsible for the formation of the ion channel structure. The β subunit is responsible for the clustering of GlyRs at the synapses through interaction of its intracellular loop with the scaffold protein gephyrin (Burgos, Yevenes, & Aguayo, 2016; Legendre, 2001; Patrizio, Renner, Pizzarelli, Triller, & Specht, 2017). GlyRs subunit expression and composition differ between brain regions and developmental age (Avila, Vidal, et al., 2013; Burgos et al., 2016; Legendre, 2001). The α2 subunit of GlyRs is highly expressed during embryonic development in the spinal cord, brainstem, cortex, hippocampus, and thalamus. However, its expression is extensively reduced during post-embryonic stages of development, especially in the spinal cord and brainstem (Avila, Nguyen, & Rigo, 2013; Avila, Vidal, et al., 2013). In contrast, α1 and α3 expressions are low during early development and newborn animals, and the expression increases significantly in the adult nervous system (Avila, Nguyen, & Rigo, 2013; Legendre, 2001; Patrizio et al., 2017). In addition, a decrease in GlyR α1 and α2 subunit protein levels, and a significant reduction in [^3^H] strychnine binding in the dorsal cochlear nucleus have been found in aged rats (28–33 months old) (H. Wang et al., 2009). However, little is known about the role of aging in GlyRs function and composition, and this is important because they are highly sensitive to ethanol, and they play a role in sedation and drinking.

### GABA_A_ receptors

The presence of the inhibitory GABA_A_R in nAc neurons has been demonstrated. Furthermore, it is well known that accumbal GABA_A_s regulate dopamine release in the nAc (Pitman et al., 2014), thus playing an important role in ethanol-addictive and feeding behavior (Basso & Kelley, 1999; Ding, Ingraham, Rodd, & McBride, 2015; Rassnick et al., 1992). Age-dependent changes in GABA_A_R expression have been extensively studied in regions such as the hippocampus and the auditory system (Caspary et al., 1999; Gahring et al., 2005; Rissman, De Blas, & Armstrong, 2007). However, few studies have examined GABA_A_Rs alterations during aging. For instance, one study shows that [^3^H] GABA binding was unchanged in the nAc of aged Sprague Dawley rats (24–30 months), suggesting that accumbal GABA_A_Rs are not overtly altered by aging (Govoni, Memo, Saiani, Spano, & Trabucchi, 1980). Nevertheless, binding studies carried out in other brain regions reported reductions in GABA_A_ receptor binding affinity in aged animals, while others reported no alterations (Govoni et al., 1980; Rissman et al., 2007). The apparent conserved global expression of GABA_A_ receptors with age could be explained by a wide variety of receptor subunits that have not been examined yet. For instance, the reduction in a particular GABA_A_ receptor subunit during aging could be compensated for by the increased expression of other subunits (Rissman & Mobley, 2011).

### Age-related changes in synaptic plasticity of accumbal MSNs

Synaptic plasticity refers to the activity-dependent modification in the strength or efficacy of synaptic transmission at the synapse (Citri & Malenka, 2008). There are several forms of synaptic plasticity, and at least two have been described in the nAc: long-term potentiation (LTP), and long-term depression (LTD) (Hoffman, Oz, Caulder, & Lupica, 2003; Ronesi & Lovinger, 2005). Moreover, the most prominent and widely studied form of accumbal plasticity is the NMDARs-dependent LTD that involves AMPARs internalization (Renteria et al., 2018; Renteria, Maier, Buske, & Morrisett, 2017). Importantly, one study showed that LTD was not altered in the dorsal striatum of aged C57BL/6J mice (17–23 months), while it was significantly reduced or absent in the nAc (Wang, 2008), supporting the idea of distinct regulations. These results are consistent with the decrease in NMDARs expression in the nAc reported in previous studies (Nicolle, Bizon, & Gallagher, 1996; Ossowska et al., 2001). Since long-term synaptic plasticity is believed to be a major mechanism for learning and memory and addictive behaviors in the nAc (Fusi, Drew, & Abbott, 2005; Kandel, 2001; Kauer & Malenka, 2007), LTD reduction in accumbal MSNs could play a key factor in the alteration of cognitive functions, motor functions, and addictive behaviors seen during aging (Menecier & Fernandez, 2012; Wolter, 2018).

### Age-related changes in electrophysiological properties and neuronal excitability in accumbal MSNs

Changes in neuronal excitability in the nAc during aging have not been studied in detail. An electrophysiological study in neurons from the dorsal striatum of young (3–5 months) and elderly (24–26 months) Fischer 344 rats indicated that aging leads to a decrease in MSNs excitability (Cepeda et al., 1992). Furthermore, this study also demonstrated that properties such as action potential amplitude, rise time and duration, resting membrane potential, input resistance, and time constant were unchanged in older animals. However, these results need to be expanded because MSNs display different properties in ventral and dorsal areas (Taverna, Canciani, & Pennartz, 2007; Willett et al., 2016). Although the effects of aging on accumbal MSN have not been studied, it was reported that the binding potential of D2R decreases in the nAc of aged individuals (Dang et al., 2017; Giacobbo et al., 2022). Consequently, alterations in D2R might affect the excitability of nAc neurons through modulation of voltage-sensitive sodium currents by Ca^2+^ signaling and modulation of K^+^ channels (Hu, Dong, Zhang, & White, 2005; Perez, White, & Hu, 2006). Is it possible that an age-related decline in the function of dopamine receptors in the nAc could alter the neuronal excitability of MSNs? Will the passive properties of accumbal neurons remain unaltered with aging? These and other questions remain to be answered.

### Changes in mesolimbic-related human behaviors

The premise of neurobiological research is that it serves to understand human pathology and translate it into new biomedical therapies. Therefore, here we attempt to relate the previous experimental data with human evidence. *In vivo* functional magnetic resonance imaging (fMRI) studies in the elderly have indicated that the nAc shows specific regional atrophy with relevant functional implications (Boisgontier et al., 2016). Therefore, in this section, we will review evidence implicating the changes mentioned above. Together, these alterations modify the neurobiological response of the nAc to processes such as reward, learning, decision-making, and addictive behaviors.

#### Reward.

Healthy aging has been associated with reduced performance in reward-related learning (Dreher et al., 2008; Mell et al., 2009; Rademacher, Salama, Gründer, & Spreckelmeyer, 2014; Schott et al., 2007). However, it is still not well understood how reward-related behavior is affected during normal aging (Rademacher et al., 2014). Studies using event-related functional magnetic resonance imaging (fMRI) showed a reduced activation of the ventral striatum for a conditioned stimulus that predicted monetary reward in old individuals when compared with young adults, suggesting that healthy elderly might be less competent in learning the predictive value of reward signals (Dreher et al., 2008; Schott et al., 2007). Consistently, a study that combined functional neuroimaging with a dynamic financial investment task demonstrated that older adults made more suboptimal financial decisions than young adults. This age-related effect was attributed to temporal variability in the neural activity of the nAc, which might be associated with reduced dopaminergic tone (Samanez-Larkin et al., 2010). In contrast, Samanez-Larkin et al. (2007) found no age-related differences in nAc activity during gain anticipation (Samanez-Larkin et al., 2007). The divergences in these findings might be partially explained by the fact that some of these studies incorporated learning more complex components to the presented task. Thus, it seems that aging differences are larger in those studies that included learning in the assignment (Dreher et al., 2008; Mell et al., 2009; Rademacher et al., 2014; Samanez-Larkin et al., 2007; Schott et al., 2007). Furthermore, Rademacher et al. (2014) suggest that aging differences in nAc activity may be modulated by the type of expected reward. For instance, event-related fMRI detected enhanced activation of the nAc to cues of social reward in older individuals, whereas increased activity to cues of monetary reward was observed in younger individuals (Rademacher et al., 2014). Consequently, these data support the notion that older adults have reduced nAc activity during decision-making based on reward, suggesting an altered stimulus-reward learning during aging. All these changes could be interpreted as resulting from changes in dopamine signaling and accumbal alterations.

#### Action selection times.

Another functional change that has been attributed to nAc atrophy in older adults is the ability to respond to complex tasks. Specifically, action selection times in the face of a complex executive task were longer in aging adults. Moreover, atrophy of the left nAc was predictive in more complex task conditions in older individuals as compared to young individuals, suggesting that the importance of the nAc for the process of action selection is incremented with age (Boisgontier et al., 2016).

#### Taste neophobia.

The role of the nAc in learning aversion to taste and in processing palatability of taste has been demonstrated (Fenu, Bassareo, & Di Chiara, 2001; Pedroza-Llinás, Ramírez-Lugo, Guzmán-Ramos, Zavala-Vega, & Bermúdez-Rattoni, 2009; Ramirez-Lugo, Nunez-Jaramillo, & Bermudez-Rattoni, 2007). Recent studies indicated that older adults have attenuation of taste neophobia (Grau-Perales et al., 2019). This alteration was demonstrated in the nAc of adult and aged rats by immunohistochemistry of cFOS (a marker of neuronal activation). An increase in immunostaining in cFOS was associated with an attenuation in taste neophobia in response to a new flavored solution (Morón & Gallo, 2007). These alterations are probably associated with a decrease in dopamine receptors that participate in the conditioned learning of aversion to taste.

### Actions of ethanol on critical synaptic and excitability-related targets in accumbens in young animal models

#### Accumbal dopamine release and ethanol

A study using an optogenetic approach to increase dopamine levels in the nAc suggested that tonic stimulation of VTA dopaminergic cells attenuated ethanol drinking behavior in rats (Bass et al., 2013). Moreover, other studies demonstrated a relation between ethanol consumption and reduced expression of the dopamine transporter (DAT) in the nAc of young animals (~25 g) (Bahi, 2020; Bahi & Dreyer, 2019; Yoshimoto et al., 2000). DAT knockdown in the nAc of mice decreases voluntary ethanol drinking and attenuated acquisition of ethanol-induced conditioned place preference (CPP) (Bahi, 2020; Bahi & Dreyer, 2019), whereas DAT overexpression exacerbated ethanol-induced CPP (Bahi, 2020). Additionally, knockdown of D1R mRNA, as well as pharmacologic inhibition of D1R in the nAc, reduced ethanol intake and decreased ethanol CPP acquisition. Meanwhile, inhibition of D2R did not affect ethanol-induced conditioned place preference, suggesting a key role of D1R in ethanol rewarding properties (Bahi & Dreyer, 2012; Young, Dreumont, & Cunningham, 2014).

#### Effects of ethanol on main brain neurotransmissions

Some evidence indicate that ethanol can interact with different neurotransmitter receptors in the central nervous system such as GABA_A_Rs, GlyRs, NMDAs, and AMPARs, among others (Abrahao et al., 2013; Aguayo et al., 2014; Burgos, Muñoz, Guzman, & Aguayo, 2015; Jonsson, Ericson, & Söderpalm, 2014; Kumar et al., 2009; Lovinger, 1993). Thus, chronic and acute ethanol exposure induces alterations in central neurotransmitter function, which is associated with abnormal neuroplasticity and neuropathology (Alhaddad et al., 2020; Bauer et al., 2013; Dahchour & Ward, 2022; Gu et al., 2013; Kapasova & Szumlinski, 2008; Mishra & Chergui, 2013). Studies in animals and humans have revealed significant increases in extracellular glutamate in the nAc after ethanol intoxication and withdrawal, and manipulation of glutamate concentrations affects ethanol drinking behavior in rodents (Bauer et al., 2013; Griffin et al., 2014; Griffin, Ramachandra, Knackstedt, & Becker, 2015; Gu et al., 2013). In contrast, electrophysiological experiments in the nAc demonstrated that acute treatment with high concentrations of ethanol (50 mM) decreases glutamatergic neurotransmission through the activation of presynaptic GABA_A_ and GABA_B_ receptors in adolescent mice (22–30 days old) (Mishra & Chergui, 2013).

#### Effects of ethanol on passive membrane properties

The activity of MSNs is regulated by electrical membrane properties, and dysregulation of these properties has been proposed as an important neuroadaptation underlying addiction (Marty & Spigelman, 2012a). In addition, ethanol regulates the activity of several voltage- and ligand-gated ion channels including voltage-dependent Na^+^, Ca^2+^, and K^+^ channels, which are involved in the control of action potential and consequently neuronal activity (Gallegos et al., 2019; Katsura et al., 2006; Kim & Suh, 2021; Marty & Spigelman, 2012a; Morton & Valenzuela, 2016; Xiao et al., 2008; Zucca & Valenzuela, 2010). Studies revealed that acute treatment with low doses of ethanol significantly decreased action potential firing in MSNs from the nAc after applying a depolarizing current pulse in 2-month-old mice (Gallegos et al., 2019; Muñoz et al., 2020). Furthermore, chronic intermittent ethanol treatment induces long-lasting alterations in membrane properties consistent with an increase in the inward rectifier and A-type K^+^ currents in MSNs from the nAc (Marty & Spigelman, 2012b). Importantly, the data indicate that distinct mouse models with a genetic predisposition for elevated alcohol intake have variations in the expression of mRNA that encode different voltage-gated channels (Mulligan et al., 2006, 2011). K^+^ channels, for instance, are key modulators of neuronal activity, and their activation leads to K^+^ efflux that reduces neuronal excitability (Dopico, Bukiya, & Martin, 2014). Importantly, different studies have shown that acute exposure to alcohol modulates several K^+^ channels (Bukiya et al., 2014; Dopico et al., 2014; Kim & Suh, 2021; Koyama, Brodie, & Appel, 2007; Kuntamallappanavar & Dopico, 2016; Liu, Asuncion-Chin, Liu, & Dopico, 2006; Liu, Vaithianathan, Manivannan, Parrill, & Dopico, 2008; Marty & Spigelman, 2012b). For example, ethanol inhibited voltage-gated K^+^ (Kv7) channels by reducing the open probability and its PI(4,5)P2 sensitivity (Kim & Suh, 2021). On the other hand, ethanol increased spontaneous firing frequency in dopaminergic VTA neurons, which was correlated with ethanol inhibition of Kv7 channels (Koyama et al., 2007). Studies demonstrated that high conductance Ca^2+^ and voltage-gated K^+^ (BK) channels could be modulated differentially, either activated or inhibited, by ethanol (Kuntamallappanavar & Dopico, 2016; Liu et al., 2006, 2008). Moreover, work by Kuntamallappanavar and Dopico (2016) suggests that different β subunits are responsible for different ethanol responses of BK channels, under identical recording conditions (Kuntamallappanavar & Dopico, 2016). Meanwhile, studies in hippocampal neurons indicate that L-type voltage-gated Ca^2+^ channels are inhibited by acute ethanol treatment, whereas chronic ethanol exposure induces upregulation of these channels (Katsura et al., 2006; Morton & Valenzuela, 2016; Zucca & Valenzuela, 2010). Finally, regarding Na^+^ voltage-gated channels, some data indicate that alcohol suppresses the activity of these channels, thus contributing to the inhibitory effect of ethanol in neurons (Horishita & Harris, 2008; Xiao et al., 2008). In summary, these studies suggest an important role of voltage-gated ion channels in ethanol consumption and addictive behaviors.

### Effect of ethanol on LGICs

#### Ethanol on ionotropic glutamate receptors (iGluRs) and synaptic plasticity

It is well known that MSNs in the nAc receive strong glutamatergic input from cortical, hippocampal, and amygdaloid structures (De Leonibus et al., 2003; Zhang, Hendricson, & Morrisett, 2005). Furthermore, fast excitatory synaptic transmission in the nAc occurs mainly through AMPA and NMDA receptors, and both receptors play a critical role in reward-seeking and addictive behaviors (De Leonibus et al., 2003; Giertler, Bohn, & Hauber, 2005; Turner et al., 2018). Several studies demonstrated that acute and chronic ethanol treatment inhibits NMDARs and modulates both expression and function of the receptor as well as its selective subunits (Abrahao et al., 2013; Jeanes, Buske, & Morrisett, 2014; Lovinger & Roberto, 2013; Nona, Li, & Nobrega, 2014; Zamudio, Smothers, Homanics, & Woodward, 2020). In addition, NMDARs are important regulators of ethanol dependence, relapse, withdrawal, and craving (Krystal, Petrakis, Mason, Trevisan, & D’Souza, 2003; Trujillo & Akil, 1995). Moreover, several studies showed that intoxicating concentrations of acute ethanol treatment inhibit NMDARs (Chu, Anantharam, & Treistman, 1995; den Hartog et al., 2013; Lovinger, White, & Weight, 1990; Wirkner, Eberts, Poelchen, Allgaier, & Illes, 2000; Zamudio, Gioia, Lopez, Homanics, & Woodward, 2020; Zamudio, Smothers, et al., 2020), and site-directed mutagenesis identified specific domains in GluN1 and GluN2 NMDAR subunits that influence ethanol inhibition of these receptors (Honse, Ren, Lipsky, & Peoples, 2004; Ronald, Mirshahi, & Woodward, 2001; Zamudio, Smothers, et al., 2020). For instance, knock-in containing mutated ethanol-resistant GLuN2A subunits showed reduced sensitivity to the sedative and motor-coordinating effect of ethanol when compared to wild-type animals. In contrast, no effect of the GluN2A mutation on ethanol-induced voluntary drinking was observed (Zamudio, Smothers, et al., 2020). In addition, GluN2A and Glun1A ethanol-resistant knock-in male mice had reduced or delayed scalation in ethanol drinking after chronic intermittent ethanol vapor exposure, suggesting that these NMDAR subunits play a critical role in ethanol dependence (Zamudio, Gioia, et al., 2020). Consistently, chronic ethanol exposure induces alterations in NMDARs expression, function, and subcellular localization (Abrahao et al., 2013; Chandler, Norwood, & Sutton, 1999; Nagy, 2008; Wang et al., 2010). Although less is known about ethanol in AMPARs, studies in cultured neurons showed that these receptors are inhibited by ethanol at similar concentrations as NMDARs (Lovinger, 1993; Wirkner et al., 2000). Moreover, withdrawal after chronic ethanol exposure increased AMPARs function and expression in several brain regions (Brückner, Rossner, & Arendt, 1997; Chandler et al., 1999; Haugbøl, Ebert, & Ulrichsen, 2005; Netzeband, Trotter, Caguioa, & Gruol, 1999). However, the mechanisms underlying the alterations in NMDARs and AMPARs function are still under investigation.

It is well known that young female mice (2–3 months) drink more than male mice (Hilderbrand & Lasek, 2018; Yoneyama, Crabbe, Ford, Murillo, & Finn, 2008), and recent studies in humans demonstrated higher at-risk alcohol consumption in middle-aged and aged women (Ahlner et al., 2018; Dare et al., 2020). Furthermore, sex differences in the behavioral response to ethanol were observed in aged rats. For instance, aged females, but not males, exhibited social facilitation after intraperitoneal injection of low doses of ethanol (Perkins et al., 2018). However, there are no available data demonstrating sex differences in alcohol intake in older animals and even less about underlying mechanisms.

Several studies have demonstrated that drugs of abuse disrupt synaptic plasticity in the mesolimbic system by remodeling dendritic spines and impairing LTD (Abrahao et al., 2013; Alhaddad et al., 2020; Jeanes et al., 2011; Lüscher & Malenka, 2011; Olsen, 2011; Ostroumov & Dani, 2018; Renteria et al., 2017, 2018). In addition, studies using D1DR-GFP and D1DR-Tomato transgenic mice demonstrated that NMDA-dependent LTD is present in D1R-expressing MSNs in the nAc shell in ethanol-naïve mice, whereas in MSNs presumably expressing D2R (D1R negative), LTP is the main form of plasticity in naïve mice (Ji et al., 2017; Renteria et al., 2017, 2018). Chronic ethanol exposure inhibited the expression of NMDA-dependent LTD in D1R MSNs neurons from the nAc shell (Jeanes et al., 2014; Renteria et al., 2017, 2018). Additionally, drinking in the dark (DID) experiments showed that after 2 weeks of daily alcohol binges, MSNs expressing D1R from the nAc core shifted from LTD to LTP, and LTP was inhibited in D1R negative neurons. Interestingly, the effect of DID in D1R expressing MSNs was reversed by pharmacological inhibition of this receptor (Ji et al., 2017). Furthermore, studies of ethanol-induced locomotor sensitization showed that high-sensitized mice increased their alcohol consumption, had a reduced accumbal NMDA receptor expression and function, and had a deficit in NMDA-dependent LTD in the nAc (Abrahao et al., 2013; Nona et al., 2014). On the other hand, low-sensitized mice exhibited an increase in NMDAR expression (Nona et al., 2014). Also, chronic ethanol consumption increased the expression of BDNF (involved in synaptic plasticity) in MSNs of the nAc shell and resulted in the loss of dendritic spines, a decrease in tyrosine hydroxylase immunostaining, and impaired LTD formation (Alhaddad et al., 2020; Spiga et al., 2014). Thus, these studies in young animals (1–3 months) suggest that altered glutamatergic synaptic plasticity is a main neurobiological component in the development of ethanol addictive behavior.

### Inhibitory LGICs

Several studies have reported the presence of GlyRs in the nAc (Forstera et al., 2017; Muñoz et al., 2018). Analysis of mRNA expression levels and immunoreactivity of GlyRs subunits have shown that α1, α2, α3, and β are expressed in the nAc (Forstera et al., 2017). Pharmacological and behavioral studies in 2–3-month-old rodents suggest that GlyRs could be involved in regulating nAc dopamine levels and ethanol addictive behaviors (Molander & Söderpalm, 2005a, 2005b; Muñoz et al., 2020). Studies performed in rats showed that local nAc perfusion of the GlyRs antagonist strychnine reduced accumbal dopamine output, whereas local addition of the GlyRs agonist glycine increased dopamine levels in the nAc (Jonsson, Adermark, Ericson, & Söderpalm, 2014; Lidö, Ericson, Marston, & Söderpalm, 2011; Molander & Söderpalm,2005a, 2005b). On the other hand, pretreatment with strychnine inhibited the effect of ethanol in nAc dopamine levels (Clarke, Söderpalm, Lotfi, Ericson, & Adermark, 2015; Jonsson, Adermark, Ericson, & Söderpalm, 2014; Lidö et al., 2011; Lidö, Stomberg, Fagerberg, Ericson, & Söderpalm, 2009; Molander, Löf, Stomberg, Ericson, & Söderpalm, 2005; Molander & Söderpalm, 2005a, 2005b). In addition, it is widely recognized that ethanol modulates non-synaptic GlyRs in the nAc, thus incrementing GlyRs-mediated tonic inhibition (Muñoz et al., 2020).

Recent work showed that α1 and α2 knock-in (KI) mice (2 months old), with normal glycinergic functions in the nAc despite the presence of ethanol-insensitive GlyRs, showed higher intake of ethanol upon first exposure, rather than the gradual consumption observed in wild-type animals (Gallegos et al., 2021; Muñoz et al., 2020). Although more complex in scope and interpretation because of compensations, α2 and α3 KO mice also displayed an alteration in ethanol intake, suggesting that these receptors play an important role in the control of reward (San Martin et al., 2020; San Martin et al., 2021). These data strongly support a key role of GlyRs in reward network excitability. However, while the association between GlyRs and ethanol sensitivity has been found, much less is known about how this glycinergic pathway specifically regulates aspects of reward processing and nAc function.

Data suggest that GABA_A_Rs are an important target for alcohol in the nAc and might be involved in voluntary ethanol consumption (Kumar et al., 2009; Leggio et al., 2019). These receptors contain a variety of subunits leading to the formation of multiple isoforms that probably differ in their ethanol sensitivity and extrasynaptic or synaptic localization (Kumar et al., 2009; Leggio et al., 2019). For instance, studies in rodents (2–3 months old) showed that increased expression or function of the α6 GABA_A_ subunit is related to reduced alcohol intake and increased GABAergic inhibition in the nAc (Leggio et al., 2019; Saba et al., 2001). On the contrary, knockdown of the GABA_A_R α4 subunit in the nAc shell reduced voluntary intake and level press responding to ethanol, but not to sucrose (Rewal et al., 2009, 2012). Several studies have shown that low concentrations of ethanol act through extrasynaptic δ-containing GABA_A_Rs to increase tonic inhibition, and α6βδ and α4βδ GABARs at extrasynaptic sites have been suggested to be sensitive to moderate concentrations of alcohol (Nie, Rewal, Gill, Ron, & Janak, 2011; Olsen, 2011; Wallner, Hanchar, & Olsen, 2003). Additionally, viral-mediated GABA_A_R δ-subunit knockdown in the nAc shell in rats decreased alcohol intake (Nie et al., 2011). These results suggest that extrasynaptic GABA_A_Rs in the nAc shell might contribute to the voluntary consumption of moderate levels of alcohol (Nie et al., 2011; Rewal et al., 2012). Additionally, intermittent ethanol treatment (CIE) and withdrawal (40 days) decreased ethanol potentiation of extrasynaptic GABA_A_Rs, while increasing potentiation of synaptic receptors. Furthermore, analysis of GABA_A_R surface subunit levels by western blot showed a diminution in α1 and δ, and an increment in α4, α5, and γ2 after CIE and withdrawal (Liang et al., 2014). Therefore, the effect of ethanol in GABA_A_R might differ with subunit composition and synaptic localization (Clarke et al., 2015; Kumar et al., 2009; Liang et al., 2014; Olsen, 2011). Intriguingly, the GABA_A_R antagonist bicuculline increases dopamine levels in the nAc. Thus, while both glycinergic and GABAergic systems are inhibitory, they seem to exert an opposite effect on nAc dopamine levels (Clarke et al., 2015). In conclusion, ethanol affects a number of critical molecular targets in relatively young animals.

### Effects of ethanol on aged targets in the nAc and other brain regions

Having examined a number of critical brain targets that are very sensitive to ethanol and influence ethanol-induced behaviors, the question is: How are they affected by ethanol when they are expressed in an aged brain? This question is relevant because the world population is not only aging but is showing more drinking problems together with increased sedation. Unfortunately, there are insufficient studies that have dealt with this question.

As reviewed in the previous sections, ethanol differentially affects young and aged human brains. In addition, from a number of studies, mostly in young animal models, it is evident that ethanol affects various membrane proteins, both ion channels and neurotransmitter receptors. However, very few studies have examined the effects of ethanol on these targets in the aged brain, especially in mesolimbic regions.

**Fig. 2.**
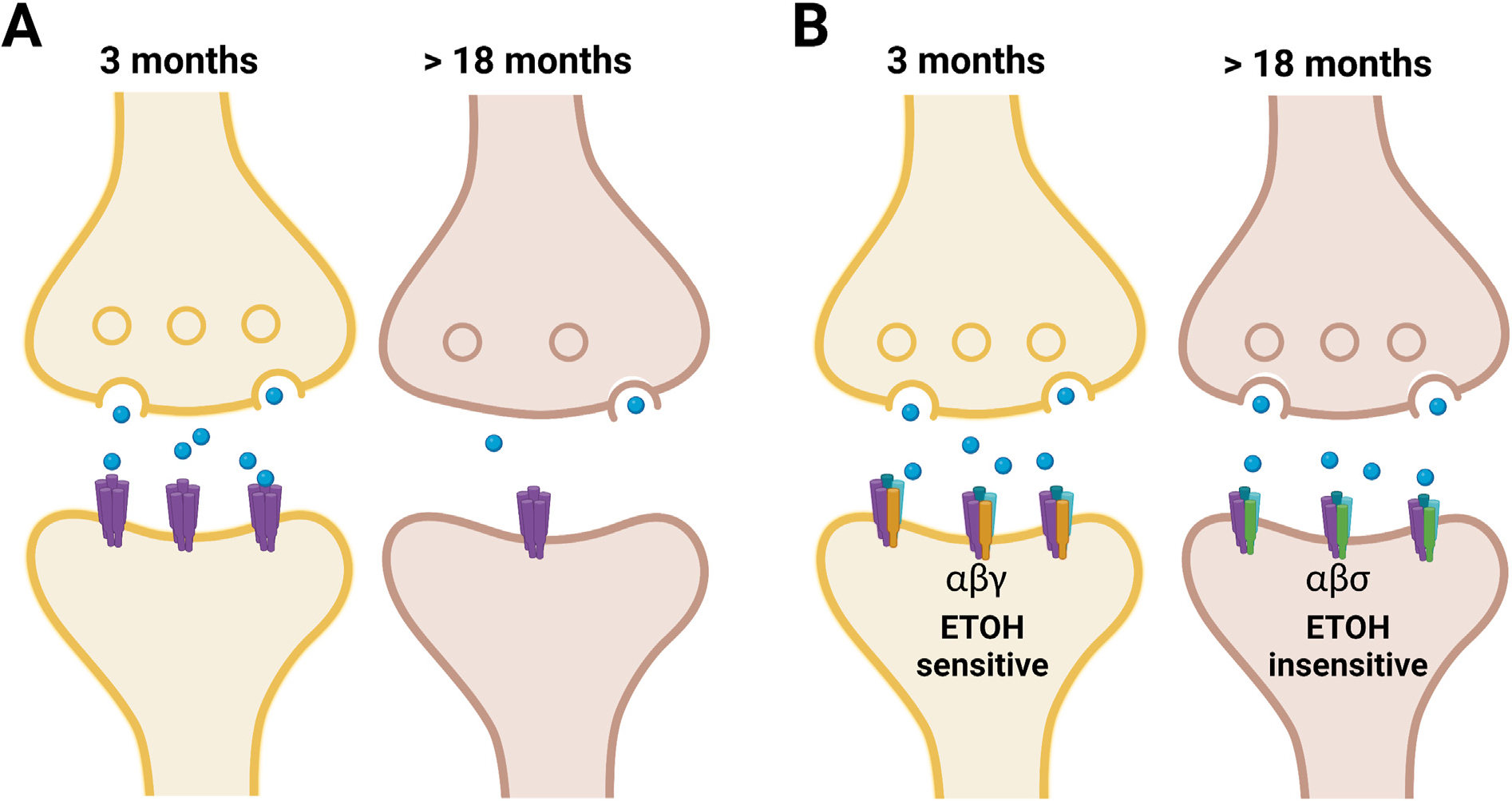
Potential synaptic modifications that alter ethanol depressive and/or rewarding effects during aging. (**A**) Weakened synaptic transmission following decreased synaptic functions. (**B**) Changes in receptor conformation (subunits) lead to alterations in ethanol sensitivity. Created with BioRender.com.

For example, data from striatal neurons recorded in young (3–5 months old), middle-aged (10–12 months), and old (greater than 24 months old) rats showed that neuronal excitability induced by monosynaptic stimulation was reduced with age (Cepeda, Walsh, Hull, Buchwald, & Levine, 1989). On the other hand, the properties of action potentials and resting membrane potentials were unchanged by age, suggesting that changes with aging are related to synaptic modifications as described above. Therefore, the changes reported in ethanol-induced behaviors with age might be due to: 1) weakening in the synaptic transmission making the brain more sensitive to the depressing action of ethanol, and/or 2) changing the sensitivity of the target to ethanol and altering its depressive or rewarding effects (see Fig. 2).

NMDAR and AMPAR have been extensively studied and are key regulators of synaptic plasticity (Henley & Wilkinson, 2013; Jurado, 2017). In the hippocampus and cortex, these receptors are significantly less functional with aging, even causing a reduction in dopamine release in the nAc (A. Kumar & Foster, 2019; Pandey et al., 2015; Segovia & Mora, 2005). Also, their reduced function likely accounts for age-related decreases in cognitive functions (A. Kumar & Foster, 2019). Nevertheless, few studies have examined changes in iGluRs in the nAc during aging. On the other hand, recent work showed that protein levels of GABA_A_Rs (α1, β3, and γ2) were not affected in the old human brain (Pandya et al., 2019). Interestingly, a lack of changes with aging was also found in mouse hippocampus, and the expression of most subunits was not affected even at 24 months (Palpagama et al., 2019). No reports of ethanol on older neurons are available.

At the presynaptic site, the release of GABA from brain synaptosomes was inhibited more significantly by ethanol in young mice than in older mice. The data showed that the IC_50_ for ethanol was significantly lower in younger mice than the older mice. The effect was also dependent on the time of administration. GABA release in the presence of ethanol was affected by chronic ethanol administration. In ethanol-tolerant young mice, the inhibition of release was significantly less as compared with young control animals. Interestingly, no inhibition was observed in older mice, and these effects were interpreted as indicating that membranes from aged animals were less disordered by ethanol (Strong & Wood, 1984).

**Fig. 3.**
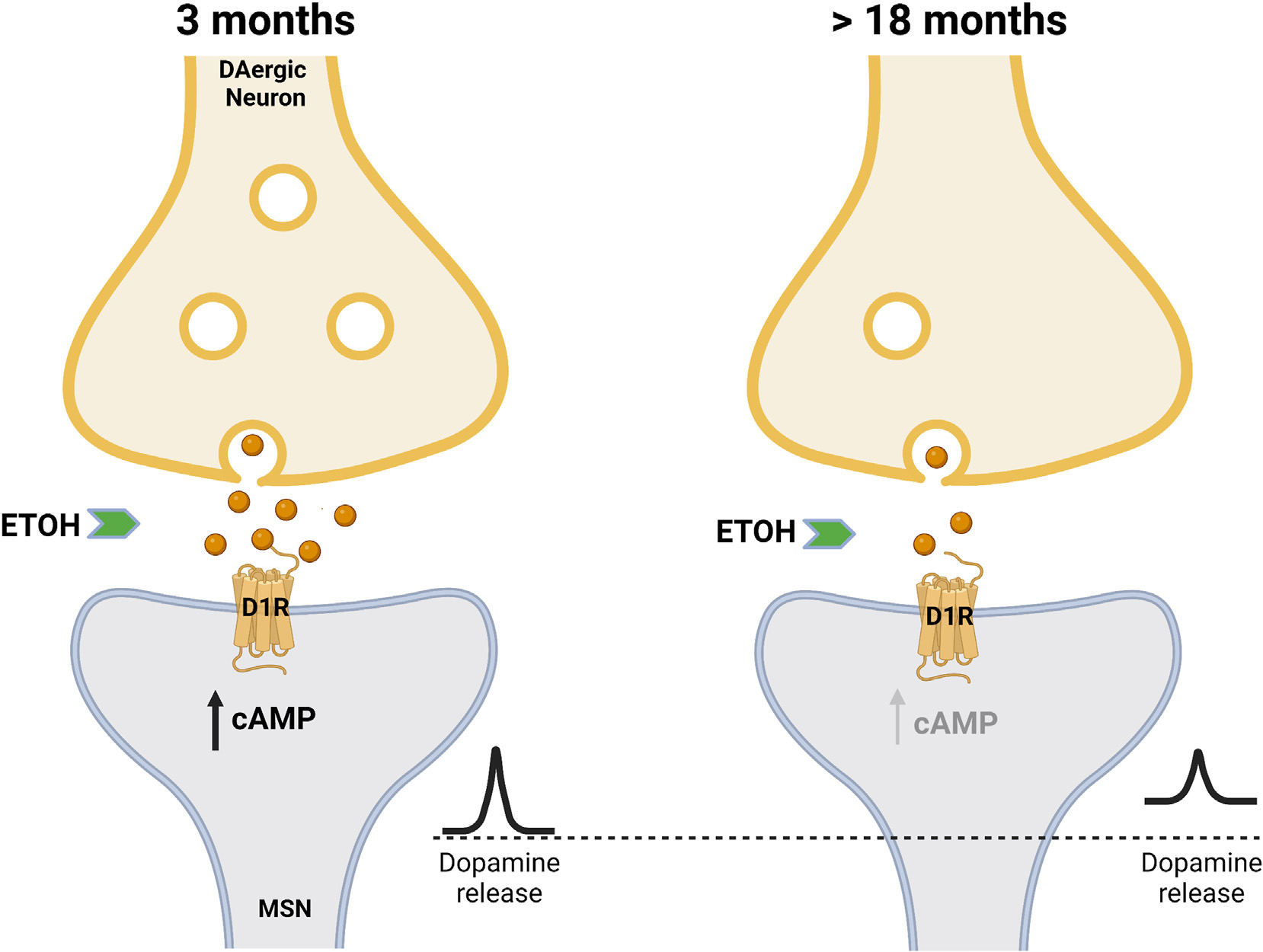
Effects of ethanol in DA levels in young and aged nAc. Acute ethanol increases DA levels in the nAc in young individuals leading to D1R containing MSNs activation by a cAMP-dependent mechanism. Ethanol might cause a smaller DA release in older nAc albeit presenting a higher level of basal dopamine. These might explain changes in ethanol-induced behaviors in aging. Created with BioRender.com.

On the other hand, ethanol applied to the nAc caused a smaller release of DA and 5-HT in older rats compared to younger rats (14 vs. 4 months old). Overall, younger neurons showed a higher sensitivity to ethanol than older neurons. Interestingly, the basal release of DA and 5-HT in the nAc was higher in older animals (Yoshimoto et al., 1998). In another study, aging was associated with a reduced level of DA in the striatum and ventral tegmental area (Woods & Druse, 1996). This study also showed that a 6-week ethanol exposure reduced DA in the VTA of 5-month-old rats, an effect that was comparable in size to that detected in control aged (24-month-old) rats. A similar reduction in dopamine level was observed in alcohol-preferring Alko alcohol rats in the dorsal cortex and striatum (Jaatinen et al., 2013) (see Fig. 3).

On the other hand, the effects of aging or ethanol on GlyRs, another critical inhibitory LGIC, has not been examined, and only one study in the dorsal cochlear nucleus reported a decrease in GlyRs α1 and α2 levels in 28–33-month-old rats (Wang et al., 2009). Also, a reduction in GlyRs (Fernandez-Perez et al., 2020), but not in GABA_A_R, was reported together with gephyrin that correlated with a decrease in the glycinergic current in slices and dissociated MSNs in a pathological aging model (Fernandez-Perez et al., 2020). The examination of GlyRs function and conformation in aging is important because they are highly sensitive to ethanol, and they play a role in sedation and drinking in young animals (Gallegos et al., 2021; Muñoz et al., 2020). Therefore, changes in their expression in the aging nAc might have a critical impact on ethanol drinking and preference, and future studies should examine these possibilities.

The review of the available data underscores the limited information on the effects of ethanol on aged mice and rats, and that many studies had considered 2–3-month-old mice as adults (Bocarsly et al., 2019; Ji et al., 2017; Muñoz et al., 2020; Patton et al., 2019). This might not be justified because 3-month-old mice are in the boundary of adolescence-adulthood, and 3–6 months might correspond to young adults, and >18 months is considered an old mouse (Flurkey, Brandvain, et al., 2007; Jackson et al., 2017). Brain maturation has been thought to be complete by 2–3 months, but as reviewed, changes are still occurring in the brain until 5–6 months of age.

## Conclusion and future directions

The global population is aging quickly, and they are increasing their ethanol consumption. They are more sensitive to the sedative actions of ethanol and binge drink more frequently with unforeseeable consequences to their health and well-being. Surprisingly, little is known about the social and biological determinants that affect drinking in the aged population. Furthermore, there is a substantial dichotomy between what we know about the effects of ethanol on neurochemical targets in young animals and how they may be affected in the aging brain. Overall, the data show that aging affects the brain by reducing connectivity and synaptic plasticity in the nAc, affecting more significantly the depressant effects of ethanol. The study of ethanol on the aging brain is an underdeveloped area, and few studies have examined the effects of ethanol in animal models more than 6 months of age.

In summary, this review focuses on the effect of aging in the nucleus accumbens and its impact on alcohol use disorders. It also provides up-to-date information on age-related morphological, molecular, and functional changes in the nAc. It also shows the main targets of ethanol in the nAc and how these could have a potential role in the increase in ethanol consumption by the elderly population recently reported. This review also shows the clear need for new studies that might contribute to understanding the targets and molecular mechanisms that regulate ethanol consumption in aged individuals. All these features justify the development of biomedical research to generate translational opportunities for new pharmacotherapies to treat alcohol abuse in the older population.

## Figures and Tables

**Table 1:** Age-associated changes in the basal concentration of dopamine, glutamate, and GABA in the nucleus accumbens. Measurement of basal concentrations of neurotransmitters in the nucleus accumbens of young and aged animals

Neurotransmitter	Levels				
	
	Young	Aged	Effect	Model	Reference

Dopamine (nM)	18.9 ± 2.7 (5 months)	11.3 ± 1.2 (24 months)	Significant	Rats	Huang et al. (1995)
	0.72 ± 0.13 (2–4 months)	0.47 ± 0.06 (27–33 months)	Significant	Rats	Segovia et al. (1999)
	0.33 ± 0.03 (2–4 months)	0.46 ± 0.06 (24–32 months)	Not significant	Rats	Segovia & Mora (2005)
Dopamine (ng/mg)	150 ng/mg (3–8 months)	100 ng/mg (22–28 months)	Significant	Mice	Winner et al. (2017)
Glutamate (μM)	0.26 ± 0.04 (2–4 months)	0.29 ± 0.05 (27–33 months)	Not significant	Rats	Segovia et al. (1999)
GABA (μM)	0.17 ± 0.01 (2–4 months)	0.14 ± 0.01 (24–32 months)	Not significant	Rats	Segovia & Mora (2005)

**Table 2:** Age-related alterations in neurotransmitter receptors in the nucleus accumbens

		Age-associated changes					
		
Receptor classes	Receptor subtype	Protein density	mRNA expression	Binding potential	Model	Method	Reference

Dopamine receptors	D1	—	—	Decreased binding potential by antagonist	Human (25–70 years old)	PET and radioligand [(11)C] SCH23390	Rieckmann et al. (2011)
	D1	—	—	No alteration in binding potential of an agonist	C57BL/6J mice (12, 18, and 24 months of age)	PET [11C]SCH23390	Giacobbo et al. (2022)
	D2	No alteration	—	—	Male Fischer 344 rats (6 and 24 months)	[3H]nemonapride binding (Autoradiography)	Araki et al. (1997)
	D2	—	—	Decreased binding potential of an agonist	Human (23 and 80 years old)	PET [18F]fallypride	Dang et al. (2017)
	D2	—	—	Decreased binding potential of an agonist	C57Bl/6J mice (12, 18, and 24 months of age)	PET [11C]Raclopride	Giacobbo et al. (2022)
	D2/D3	—	—	No alteration in binding potential of an agonist	Human (19–55 years old)	PET [11C]-(+)4-Propyl-3,4,4a,5,6,10b-hexahydro-2H-naphtho[1,2-b][1,4] oxazin-9-ol	Matuskey et al. (2016)
	D2/D3	—	—	Decreased binding potential by antagonist	Human (18–73 years old)	PET [11C]raclopride (D2/3 antagonist)	Nakajima et al. (2015)
	D3	Increased	—	—	Male Fischer 344 × Brown-Norway rats (F1) (4 and 37 months)	[3H](+)-7-hydroxy-2-(*N, N*-di-*n*-propylamino) tetralin (Autoradiography)	Wallace and Booze (1996)
	D3	—	—	No alteration in binding potential of an agonist	Human (18–73 years old)	PET [^11^C]-(+)4-Propyl-3,4,4a,5,6,10b-hexahydro-2*H*-naphtho[1,2-b][1,4]oxazin-9-ol	Nakajima et al. (2015)
Glutamate receptors	mGluR3	—	Increased (core) no alteration (shell)	—	Male Fischer 344 rats (3–25 months)	^35^S-dATP labeled hybridization	Simonyi et al. (2005)
	mGluR3	—	No alteration	—	Male Fisher 344 rats (3–25 months)	Quantitative *in situ* hybridization	Simonyi et al., 2000
	NMDA	Decreased	—	—	Female Wistar rats (3–5, 20 –21 and 29–31 months)	[H]MK-801 binding (Autoradiography)	Ossowska et al. (2001)
	NMDA	Decreased	—	—	Long—Evans Rats (4 and 24–25 months)	[3H]glutamate binding (Autoradiography)	Nicolle et al. (1996)
	AMPA	No alteration	—	—	Long—Evans Rats (4 and 24–25 months)	[H]AMPA binding (Autoradiography)	Nicolle et al. (1996)
	AMPA	Decreased	—	—	Female Wistar rats (3–5, 20 –21 and 29–31 months)	[H]AMPA binding (Autoradiography)	Ossowska et al. (2001)
GABA receptors	GABA	—	—	No alteration in agonist binding	Sprague Dawley rats (24 –30 months)	[3H]GABA binding (Autoradiography)	Govoni et al. (1980)
Acetylcholine receptors	M4	Decreased	No alteration	—	Male Wistar rats (5–6 and 24–25 months)	[^3^H]-quinuclidinyl benzilate binding (Autoradiography)	Blake et al. (1991)

Age-related alterations in the protein density, mRNA expression, and binding potential of neurotransmitter receptors in the nucleus accumbens.

D1—e3: dopamine receptor 1, 2, 3 respectively; GABA: gamma-aminobutyric acid; AMPA: α-amino-3-hydroxy-5-methyl-4-isoxazolepropionic acid; NMDA: N-methyl-D-aspartate; PET: Positron emission tomography.

Protein density or B max signifies the maximum density of receptors. This value is unique to a particular tissue in the binding assay (Dong, Liu, & Wang, 2015). Binding potential is a combined measure describing the ultimate binding force of a receptor to its ligand, denoted as the product of maximum density of a neuroreceptor (Bmax) and the affinity of a specific ligand to its receptor (Zhang & Fox, 2012).
